# Serum Bile Acid Composition in Women With Gestational Diabetes and Fasting Hyperglycaemia (HAPO): A Cohort Study

**DOI:** 10.1111/1471-0528.18363

**Published:** 2025-09-03

**Authors:** Josca M. Schoonejans, Hanns‐Ulrich Marschall, M. Martineau, David McCance, Catherine Williamson

**Affiliations:** ^1^ Institute for Reproductive and Developmental Biology Imperial College London London UK; ^2^ Department of Molecular and Clinical Medicine/Wallenberg Laboratory University of Gothenburg Gothenburg Sweden; ^3^ Regional Centre for Endocrinology and Diabetes Royal Victoria Hospital Belfast UK; ^4^ Department of Women and Children's Health King's College London London UK

**Keywords:** bile acids, diabetes in pregnancy, maternal physiology, medical disorders in pregnancy

Bile acids (BAs) are produced in the liver and released into the intestine upon feeding. Primary BAs may be de‐conjugated and converted to secondary bile acids by the gut microbiome, after which they are reabsorbed into the portal circulation. Serum BA composition changes in pregnancy, which may affect endocrine glucose regulation. In rodents, bile acid feeding and genetic manipulation of bile acid receptors affect gestational glucose tolerance [1]. We have shown that in the PRiDE study, late gestation serum BAs were increased in women with gestational diabetes mellitus (GDM) of European, but not South Asian, ethnicity, and correlated positively to fasting glucose levels [2]. This study aimed to confirm whether serum BA profiles were altered in an independent cohort of European women with GDM diagnosed based on fasting glucose.

This study used participants from the Hyperglycemia and Adverse Pregnancy Outcomes (HAPO) study (participants gave written informed consent, approved by Northern Ireland Regional Ethics Committee, RO1‐HD34242, RO1‐HD34243, RD04/0002756), who had fasting glucose in the lowest quartile (≤ 4.3 mmol/L) without GDM (*n* = 79, Control) or highest quartile (≥ 5.1 mmol/L) with GDM (*n* = 70, GDM) [3]. GDM was diagnosed if fasting plasma glucose ≥ 5.1 mmol/L and/or 2‐h plasma glucose ≥ 8.5 mmol/L during 75 g oral glucose tolerance testing. Serum BAs were measured with UPLC‐MS/MS in fasted serum collected between 25 and 31 weeks gestation (median 29 weeks). Log‐transformed BA data were analysed using multiple linear regression analysis testing for effects of GDM and BMI, corrected for maternal age.

Participants were well‐matched for gestational age and height (not shown), but women with GDM had a higher BMI (31.3 [27.7–36.2] vs. 25.2 [23.8–27.2] kg/m [2], *p* < 0.001) and age (32.1 [30.0–35.0] vs. 29.1 [25.6–33.4] years, *p* < 0.001), and the analysis was corrected for this. Total serum BAs were elevated 1.43‐fold in women with GDM (Table 1). Specifically, concentrations of individual species G‐CA, G‐CDCA, T‐CDCA, and G‐DCA were increased. Total conjugated and G‐conjugated species were significantly increased (trend for T‐conjugated species) but the conjugation ratio was not different. When combining unconjugated and conjugated species, we found an increased prevalence of the primary BA species CA (12α‐hydroxylated) and CDCA (non‐12α‐hydroxylated). Both total primary (trend) and secondary (significant) BAs were increased, but the ratio between primary and secondary BAs was unchanged. There was no difference in the CA/CDCA or 12α‐hydroxylation ratios as total 12α‐hydroxylated and non‐12α‐hydroxylated BAs were both increased. The total abundance of the most prevalent secondary BA type, DCA, was decreased with increasing BMI, resulting in an association between BMI and higher primary/secondary and lower 12α‐hydroxylation ratios.

**TABLE 1:** Concentrations of late gestation bile acids in serum from control women with low glucose and women with GDM and high fasting glucose. Bile acid concentrations shown in μmol/L: Median [interquartile range]. Individual bile acid species are shown in order of abundance. High glucose‐GDM effect: Fold‐change [95% confidence interval]. BMI effect: Fold‐change per unit BMI increase [95% confidence interval]. Significantly different bile acids are shown in bold, trends in italicised bold. *p < 0.05, **p < 0.01, ***p < 0.001, (*) 0.01 < *p* < 0.05, multiple linear regression testing for the effect of GDM and BMI corrected for maternal age.

Bile acid (μmol/L)	Control (*n* = 79)	GDM (*n* = 70)	GDM effect	BMI effect
**Total bile acids**	**1.99 [1.23–3.47]**	**2.36 [1.35–4.03]**	1.43 [1.08–1.89]*	0.98 [0.96–1.00]
**Total CA**	**0.40 [0.24–0.61]**	**0.49 [0.26–0.78]**	1.46 [1.01–2.10]*	1.01 [0.98–1.03]
***Total CDCA***	***0.34 [0.19–0.62]***	***0.37 [0.24–0.74]***	1.46 [0.99–2.14] (*)	1.01 [0.98–1.04]
Total DCA	0.75 [0.40–1.81]	0.92 [0.43–2.30]	1.54 [0.89–2.65]	0.96 [0.92–1.00]*
Total UDCA	0.03 [0.00–0.05]	0.02 [0.00–0.07]	1.08 [0.48–2.44]	1.03 [0.96–1.09]
Total LCA	0.07 [0.05–0.09]	0.08 [0.06–0.09]	1.12 [0.91–1.37]	1.00 [0.99–1.02]
***Primary***	***0.81 [0.45–1.26]***	***0.88 [0.52–1.72]***	1.32 [0.98–1.79] (*)	1.01 [0.98–1.03]
**Secondary**	**0.91 [0.49–1.86]**	**1.12 [0.56–2.51]**	1.49 [1.01–2.21]*	0.97 [0.94–1.00]*
Primary/secondary ratio	0.91 [0.49–1.86]	0.88 [0.39–2.03]	0.89 [0.56–1.41]	1.04 [1.00–1.08]*
Unconjugated	1.07 [0.55–2.44]	1.17 [0.66–2.65]	1.30 [0.89–1.92]	0.99 [0.96–1.02]
**Conjugated**	**0.65 [0.40–1.05]**	**0.88 [0.48–1.30]**	1.60 [1.22–2.11]***	0.99 [0.97–1.01]
**G‐conjugated**	**0.53 [0.34–0.84]**	**0.69 [0.40–1.08]**	1.59 [1.22–2.07]***	0.99 [0.97–1.01]
***T‐conjugated***	***0.10 [0.06–0.26]***	***0.15 [0.08–0.27]***	1.75 [0.99–3.09] (*)	0.99 [0.94–1.03]
Conjugation ratio	0.69 [0.20–1.25]	0.70 [0.32–1.39]	1.23 [0.79–1.90]	1.00 [0.96–1.03]
**12α‐hydroxylated**	**1.40 [0.76–2.93]**	**1.55 [0.92–3.29]**	1.52 [1.09–2.12]*	0.97 [0.95–1.00] (*)
**non12α‐hydroxylated**	**0.46 [0.27–0.73]**	**0.46 [0.33–0.92]**	1.31 [0.99–1.72] (*)	1.00 [0.98–1.03]
12α‐hydroxylation ratio	2.57 [1.56–6.37]	2.34 [1.58–5.43]	1.16 [0.80–1.68]	0.97 [0.94–1.00]*
CA/CDCA ratio	1.30 [0.88–1.81]	1.27 [0.82–1.66]	1.00 [0.69–1.45]	1.00 [0.97–1.03]

Individual bile acids	Control	GDM	GDM effect	BMI effect
DCA	0.53 [0.22–1.72]	0.62 [0.22–1.70]	1.73 [0.84–3.50]	0.97 [0.91–1.02]
**G‐CDCA**	**0.18 [0.09–0.32]**	**0.22 [0.14–0.45]**	1.65 [1.16–2.35]**	0.99 [0.97–1.02]
**G‐CA**	**0.15 [0.11–0.24]**	**0.20 [0.14–0.36]**	2.16 [1.45–3.21]**	0.98 [0.95–1.01]
CA	0.14 [0.09–0.32]	0.16 [0.09–0.40]	1.22 [0.68–2.21]	1.01 [0.96–1.05]
**G‐DCA**	**0.12 [0.05–0.21]**	**0.15 [0.06–0.27]**	1.85 [1.08–3.17]*	0.96 [0.92–1.00]*
CDCA	0.05 [0.00–0.16]	0.09 [0.02–0.23]	1.40 [0.52–3.75]	1.08 [1.00–1.17]*
**T‐CDCA**	**0.05 [0.02–0.10]**	**0.06 [0.03–0.10]**	2.06 [1.12–3.79]*	0.99 [0.94–1.04]
***LCA***	***0.05 [0.04–0.06]***	***0.06 [0.05–0.06]***	1.17 [0.98–1.40] (*)	0.99 [0.98–1.01]
T‐DCA	0.04 [0.00–0.08]	0.04 [0.00–0.10]	1.84 [0.80–4.22]	0.95 [0.89–1.02]
***T‐CA***	***0.02 [0.00–0.06]***	***0.04 [0.01–0.09]***	2.07 [0.92–4.67] (*)	0.99 [0.93–1.06]
G‐LCA	0.02 [0.00–0.03]	0.03 [0.00–0.03]	0.87 [0.41–1.81]	1.07 [1.01–1.13]*
G‐UDCA	0.02 [0.00–0.03]	0.02 [0.00–0.04]	1.03 [0.49–2.18]	1.02 [0.96–1.08]
T‐UDCA	0.00 [0.00–0.00]	0.00 [0.00–0.00]	1.29 [0.88–1.90]	0.99 [0.96–1.02]
UDCA	0.00 [0.00–0.00]	0.00 [0.00–0.03]	1.18 [0.53–2.65]	1.02 [0.97–1.10]
T‐LCA	0.00 [0.00–0.03]	0.00 [0.00–0.00]	1.05 [0.88–1.25]	0.99 [0.97–1.00]*

Abbreviations: BMI, body mass index; CA, cholic acid; CDCA, chenodeyxocholic acid; DCA, deoxycholic acid; G‐, glycine‐conjugated; GDM, gestational diabetes mellitus; LCA, lithocholic acid; T‐, taurine‐conjugated; UDCA, ursodeoxycholic acid.

This study confirmed our previous findings that serum BAs are increased in European women with GDM in an independent cohort, irrespective of BMI. This finding of mild (within normal range for uncomplicated pregnancy) [4] but significant elevation in BAs with GDM and fasting hyperglycaemia suggests that altered BA homeostasis may be implicated in GDM pathogenesis for some European women, or alternatively a consequence of GDM in this population. We previously showed increased levels of C4 (marker of BA synthesis) in women with GDM [2], suggesting increased hepatic production could underlie the findings in this study. Over half of women in HAPO were diagnosed with GDM based on fasting glucose [5], but studies with wider ranges of fasting glycaemia will confirm generalisability of our findings to all women with GDM. Consistent with previous work [2], increased BMI was associated with predominance of primary over secondary BAs, a finding often linked to gut microbiome changes with obesity. In this cohort, a predominance of conjugated BAs in women with GDM was observed, which is novel. This could reflect decreased de‐conjugation in the large intestine by the maternal microbiome. Further work is required to determine the cause of this compositional change.

## Author Contributions

Conceptualization: C.W. Data curation: M.M., J.M.S. Formal analysis: J.M.S. Funding acquisition: C.W. Project administration: C.W., J.M.S. Investigation: H.U.M. Resources: C.W., H.U.M., D.M.C. Supervision: C.W. Visualisation: J.M.S. Writing – original draft: J.M.S. Writing – review and editing: C.W., D.M.C., J.M.S., M.M. All individuals that qualified for authorship have been included and all those included qualify for authorship.

## Ethics Statement

This study used a subset of participants from the Hyperglycemia and Adverse Pregnancy Outcomes study approved by Northern Ireland Regional Ethics Committee (RO1‐HD34242, RO1‐HD34243, and RD04/0002756).

## Consent

Participants gave written informed consent.

## Conflicts of Interest

The authors declare no conflicts of interest.

## Data Availability

The data that support the findings of this study are available from the corresponding author upon reasonable request.
